# Effects of Microbial Inoculants on Carbon, Nitrogen, and Phosphorus Stoichiometry of Soil Aggregates

**DOI:** 10.3390/microorganisms14030583

**Published:** 2026-03-04

**Authors:** Rengui Xue, Chong Li, Xin Liu, Xuanran Yu, Ying Chen, Yue Chen, Jinchi Zhang

**Affiliations:** 1 Co-Innovation Center for Sustainable Forestry in Southern China of Jiangsu Province, Key Laboratory of Soil and Water Conservation and Ecological Restoration of Jiangsu Province, Nanjing Forestry University, Nanjing 210037, China; njfuxrg@njfu.edu.cn (R.X.); liuxinswc@njfu.edu.cn (X.L.); yuxuanran@njfu.edu.cn (X.Y.); ccchen71@njfu.edu.cn (Y.C.); 19860915971@njfu.edu.cn (Y.C.); 2 Department of Renewable Resources, University of Alberta, Edmonton, AB T6G 2E3, Canada; cli5104@njfu.edu.cn

**Keywords:** functional microbial inoculation, stoichiometry, soil–microbe–enzyme, aggregate scale

## Abstract

Functional microbial inoculation is widely applied in soil restoration; however, its effects on aggregate-scale nutrient cycling remain unclear. Based on ecological stoichiometry theory, we conducted 1-year and 3-year pot experiments using *Bacillus thuringiensis* (NL-11) and *Gongronella butleri* (NL-15) under plant-present and plant-absent conditions, with only NL-11 applied in the 1-year experiment. Aggregate size distribution, mean weight diameter (MWD), soil nutrients, microbial biomass, and enzyme activities were evaluated across aggregate classes. The results demonstrated that microbial effects were dependent on both time and plant presence. Under 3-year plant-present conditions, NL-11 and NL-15 significantly increased macroaggregate proportions and MWD, thereby enhancing aggregate stability. Under 3-year no-plant conditions, NL-15 increased organic carbon and total nitrogen in macro- and meso-aggregates by 55–59% and elevated soil C/P and N/P ratios, whereas NL-11 primarily enhanced total nitrogen. In 1-year no-plant macroaggregates, NL-11 increased microbial biomass phosphorus and reduced microbial biomass C/P and N/P ratios. Both inoculants enhanced invertase activity under plant-absent conditions, whereas plant presence stimulated acid phosphatase activity, with NAG activity increasing only under NL-15. Overall, microbial inoculation altered nutrient availability and microbial metabolic characteristics, promoted coordinated C–N–P stoichiometry, and facilitated the recovery of aggregate-scale nutrient cycling.

## 1. Introduction

Microbial inoculation introduces functional microorganisms, such as plant growth-promoting rhizobacteria and arbuscular mycorrhizal fungi, into soil or the rhizosphere to improve soil ecological functions [1,2]. Soil microorganisms act as the core drivers of nutrient cycling and participate in nearly all major soil nutrient processes [3]. By secreting extracellular enzymes, including cellulases, proteases, and phosphatases, microorganisms decompose complex organic matter into simpler inorganic nutrients, such as ammonium, phosphate, and potassium ions. They further promote carbon, nitrogen, and phosphorus cycling through processes such as carbon fixation, nitrogen fixation, and phosphorus mobilization [4,5,6,7]. Previous studies have demonstrated that microbial inoculants play an important role in regulating soil nutrients and enhancing plant growth, which explains their wide application in ecological restoration and vegetation reconstruction.

Soil aggregates are the basic units of soil structure and an important indicator of soil quality, and their stability is closely linked to nutrient storage and protection [8,9]. On the one hand, aggregates provide suitable habitats for microorganisms [10]; on the other hand, they act as nutrient “reservoirs” through physical protection [11]. Microbial hyphae contribute to aggregate formation [12], while aggregates serve as hotspots of enzyme activity that drive microscale processes of carbon, nitrogen, and phosphorus cycling [13,14]. Therefore, examining microbial inoculation effects on nutrient balance and nutrient limitation at the aggregate scale is particularly important. Ecological stoichiometry offers a theoretical framework for understanding elemental balance in ecosystems [15]. Carbon, nitrogen, and phosphorus are essential elements for life, and their stoichiometric ratios are widely used to indicate energy and nutrient limitation [16,17]. In soil nutrient cycling, complex stoichiometric relationships exist among soil, plants, microorganisms, and enzymes [18,19]. Microbial inoculation may alter microbial community structure, thereby affecting microbial biomass and its stoichiometric traits, and may further adjust enzyme allocation strategies in response to soil nutrient limitation [6]. These microbial and enzymatic changes could influence soil nutrient cycling and balance, especially over long-time scales.

Previous studies have mainly focused on the effects of microbial inoculants on plant growth and bulk soil nutrient content. These studies show that inoculation can promote plant growth, improve soil structure, and enhance soil fertility [20,21]. However, nutrient balance and nutrient limitation at the aggregate scale, as well as the long-term effects of microbial inoculation, remain poorly explored. In particular, the introduction of exogenous microorganisms may alter native microbial community structure and function. This change can affect microbial stoichiometric traits and enzyme activities [22,23], and further influence soil nutrient cycling and plant growth. The response mechanisms at the aggregate level are still unclear. Therefore, we selected *Bacillus thuringiensis* (NL-11) and *Gongronella butleri* (NL-15) as inoculants and conducted two pot experiments lasting 1 year and 3 years. Treatments included plant-present and plant-absent conditions. The aim was to evaluate how microbial inoculation affects the ecological stoichiometry of carbon, nitrogen, and phosphorus in soil aggregates of different sizes. We hypothesized that (1) the effects of microbial inoculation on soil–microbe–enzyme stoichiometric ratios are regulated by time and plant presence, and (2) different microbial inoculants show clear and specific responses at the aggregate scale.

## 2. Materials and Methods

### 2.1. Pot Experiment Design

#### 2.1.1. Experimental Material

The study was performed in a greenhouse at the Xiashu Forest Farm of Nanjing Forestry University, Xiashu Town, Jurong City, Zhenjiang, Jiangsu Province, China (32°07′09.53″ N 119°13′04.71″ E). Soil for the experiment was collected from adjacent forest land and classified as sandy loam. Soil pH, measured at a soil-to-water ratio of 1:5, was 5.22 ± 0.01. The initial contents of total carbon (TC) and total nitrogen (TN) were 3.9 ± 0.8 g·kg^−1^ and 0.6 ± 0.1 g·kg^−1^, respectively. Before use, the soil was air-dried and passed through a 5 mm sieve to remove coarse material and visible plant residues. The test plant was *Amorpha fruticosa*, which is widely used in vegetation restoration and soil rehabilitation due to its strong adaptability and tolerance to degraded soil conditions, and the seeds were purchased from Jiangsu Tianhe Nursery Co., Ltd., Yangzhou, China. Before planting, the seeds were pretreated. They were first surface-sterilized with ethanol. The seeds were then placed in sterile water at 60 °C and stirred for 5 min. After that, they were transferred to cold water and soaked for 24 h, followed by repeated rinsing with sterile water. To promote germination, the seeds were mixed with sterilized moist sand at a ratio of 1:3 and kept in a plant growth chamber at 25 °C. During this period, the seeds were covered with moist grass mats and sprayed daily with sterile water at 30 °C to maintain moisture. When the seedlings reached about 1 cm in height, they were carefully transplanted into plastic pots.

*Bacillus thuringiensis* (NL-11) [24,25] and *Gongronella butleri* (NL-15) [26] were selected for this experiment because they are plant growth-promoting microbial (PGPM) strains that have been demonstrated in previous studies to enhance plant growth and facilitate soil restoration. NL-11 was cultured on Luria–Bertani (LB) agar plates (Qingdao Haibo Biotechnology Co., Ltd., Qingdao, China) at 28 °C for 24 h. One colony was inoculated into LB liquid medium and incubated at 28 °C with shaking at 200 rpm for 48 h. The culture was transferred to a 5 L bioreactor (Sartorius BIOSTAT^®^ B Plus, Göttingen, Germany) and cultured at 28 °C for 24 h. Cells were recovered by centrifugation at 4500 rpm for 5 min. The pellet was resuspended in 0.05 M phosphate-buffered saline (PBS, pH 7.4) and adjusted to OD_600_ = 1 (approximately 1 × 10^8^ CFU mL^−1^). NL-15 was grown on rose bengal agar medium (Qingdao Haibo Biotechnology Co., Ltd., Qingdao, China) at 26 °C under dark conditions for 7 d. Mycelial plugs (5 mm in diameter) were then transferred to rose bengal liquid medium and cultured in a 5 L bioreactor (Sartorius BIOSTAT^®^ B Plus, Göttingen, Germany) at 28 °C for 120 h. After incubation, sterile saline solution (0.8% NaCl) was added to the cultures, and spores were gently scraped and collected. The suspension was filtered through sterile gauze to remove mycelial fragments. The resulting spore suspension was washed twice with sterile saline and adjusted to a final concentration of 1 × 10^7^ conidia mL^−1^.

#### 2.1.2. Experimental Design and Sampling

All soil materials, microbial inoculants, and seedlings were prepared in the same way before the experiments were set up. The whole experiment includes a long-term experiment (3 years) and a short-term experiment (1 year). The short-term experiment focused on evaluating rapid bacterial effects on soil nutrient dynamics; therefore, only NL-11 was included. The fungal inoculant NL-15 was designed in the 3-year experiment to assess longer-term responses. The long-term experiment started on 4 June 2021 and was maintained for three years. Six treatments were arranged, including NL-11 with plants, NL-11 without plants, NL-15 with plants, NL-15 without plants, a non-inoculated control with plants, and a non-inoculated control without plants. Each treatment consisted of five replicate pots. All pots were filled with 5 kg of soil. In the treatments with plants, one *A. fruticosa* seedling was transplanted into each pot. For the inoculated treatments, 60 mL of the corresponding bacterial or fungal suspension was added to each pot at the beginning of the experiment. The short-term experiment was carried out from 30 June 2023 and lasted for one year. In this experiment, four treatments were included: NL-11 with plants, NL-11 without plants, control with plants, and control without plants, with 18 replicate pots for each treatment. The experimental procedure followed that of the long-term experiment. Each pot again contained 5 kg of soil. One seedling was planted in pots assigned to the plant treatments, and 60 mL of NL-11 suspension was applied to the inoculated pots. The short-term experiment and the long-term experiment ended on 3 July 2024.

Throughout the experiment, each pot was watered weekly with 2 L of sterile distilled water. During irrigation, water was applied slowly and evenly to minimize mechanical disturbance to soil aggregates. The same watering regime was applied to all treatment groups to ensure consistent potential impacts on soil structure. Soil sampling was carried out on 3 July 2024. In treatments with plants, rhizosphere soil was collected. For the short-term experiment, every three samples were combined into one composite sample. The total number of samples from both long-term and short-term experiments was 54. All soil samples were used for subsequent aggregate fractionation and analysis of related parameters.

### 2.2. Laboratory Analyses

At the end of experiment, approximately 200 g of soil was collected from each pot after gentle homogenization to ensure representativeness. After transport to the laboratory, soil samples were processed immediately for aggregate fractionation following the method described in [27]. Air-dried intact soil was placed on the upper sieve of a sieve set consisting of 2 mm and 0.25 mm meshes arranged from top to bottom. The sieve stack was gently shaken to minimize disturbance to aggregate structure. During sieving, smaller aggregates passed through the meshes, while larger aggregates were retained on the corresponding sieves. Soil from each aggregate size fraction was collected separately, weighed, and stored in self-sealing bags. In total, 162 aggregate samples were obtained and used for subsequent analyses, including soil organic carbon (SOC), total nitrogen (TN), total phosphorus (TP), microbial biomass carbon (MBC), microbial biomass nitrogen (MBN), and microbial biomass phosphorus (MBP), as well as the activities of β-1,4-glucosidase (BG), invertase, β-1,4-N-acetylglucosaminidase (NAG), urease, and acid phosphatase (ACP).

Soil organic carbon [27] and total nitrogen [28] were measured using an elemental analyzer. Total phosphorus was determined by the molybdenum blue colorimetric method after digestion with HClO_4_–H_2_SO_4_ [29]. Microbial biomass carbon [30] and nitrogen [31] were analyzed using the chloroform fumigation–extraction method and quantified with an organic carbon analyzer. Microbial biomass phosphorus was extracted from fumigated soil with a sodium bicarbonate solution and determined by colorimetric analysis [32].

Enzyme activities were determined using standard colorimetric assays. β-1,4-glucosidase (BG) activity was measured by quantifying p-nitrophenol release [33]. Invertase activity was analyzed using the 3,5-dinitrosalicylic acid (DNS) method [34]. β-1,4-N-acetylglucosaminidase activity was assessed based on the colorimetric detection of p-nitrophenol [35]. Urease activity was measured using the phenol–sodium hypochlorite method [35], while acid phosphatase activity was determined with the disodium p-nitrophenyl phosphate colorimetric assay [34].

### 2.3. Statistical Analysis

Stoichiometric ratios of soil properties, microbial biomass, and enzyme activities were calculated from the measured variables. For enzyme activities, units were standardized and stoichiometric relationships were derived using a logarithmic ratio approach [6,36]. Because microaggregates smaller than 0.25 mm showed low recovery and resulted in substantial data loss, this fraction was excluded from statistical analyses. Linear mixed-effects models (LMMs) were used to test the effects of microbial inoculation (Inoculants), experimental duration (Time), plant presence (Plant), and aggregate size (Aggregate) on soil properties, microbial biomass, and enzyme activity variables. To assess inoculation effects under specific condition combinations (Time × Plant × Aggregate), a full four-factor interaction model was fitted and estimated marginal means were calculated. Simplified models including only main effects and two-way interactions were also fitted to allow clearer interpretation of individual factors and their interactions, and fixed effects were tested using Type III analysis of variance. All analyses were performed in R (version 4.5.1) with the lme4 (version 1.1.38) package. Model residuals were checked to assess model assumptions, and variables showing evident skewness or heteroscedasticity were log-transformed before analysis. Mean Weight Diameter (MWD) was calculated as an index of soil aggregate stability. And non-parametric Kruskal–Wallis tests and Wilcoxon rank-sum tests were employed to evaluate treatment effects within each Time × Plant combination. Relationships among soil nutrients, microbial biomass, and enzyme activities were analyzed using Spearman correlation, with calculations and figures produced in Python (version 3.13.7) using the scipy (version 1.16.2) and matplotlib (version 3.10.6) packages.

## 3. Results

### 3.1. Aggregate Size Distribution and MWD

Results from the non-parametric tests (Table S1) showed that aggregate size distribution and MWD showed no significant response to inoculation in the absence of plants (*p* > 0.05). In contrast, inoculation significantly altered soil structure under plant-present conditions. Specifically, in the 1-year experiment, NL-11 significantly affected the proportions of >2 mm (Figure S1a) and <0.25 mm (Figure S1c) aggregates as well as MWD (Figure S1d) compared to the control (*p* < 0.05). In the 3-year experiment, significant shifts in >2 mm and 0.25–2 mm (Figure S1b) fractions and MWD were observed for both NL-11 and NL-15 treatments.

### 3.2. Soil Nutrients and Their Stoichiometric Characteristics

**Figure 1:**
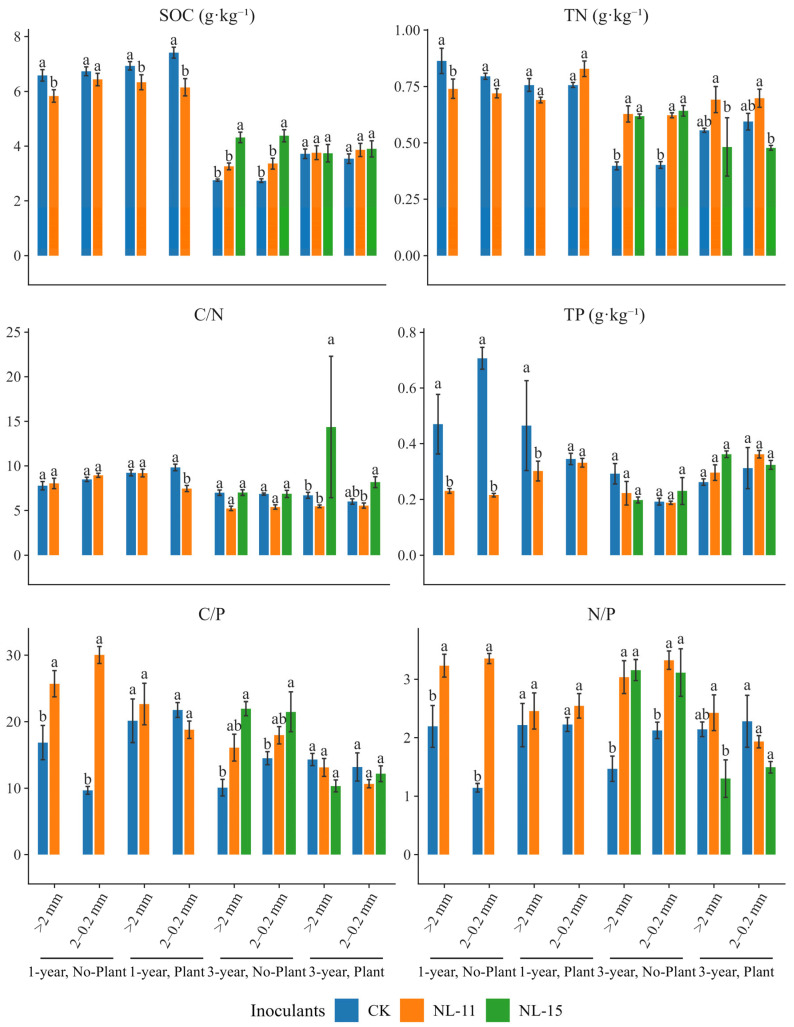
Responses of soil nutrient contents and stoichiometric ratios to microbial inoculation. Bars represent Means ± SE. Inoculants were as follows: CK, no inoculation; NL-11: treated with *Bacillus thuringiensis* inoculation; NL-15: treated with *Gongronella butleri* inoculation. Different lowercase letters indicate significant differences among inoculation treatments within the same Group (Time × Plant × Aggregate) based on Tukey-adjusted post hoc comparisons (*p* < 0.05). Percentage changes reported in the text were calculated relative to the control using estimated marginal means.

The results showed (Figure 1) that, compared with the control, NL-15 inoculation significantly increased soil organic carbon (by 56–59%), total nitrogen (by 55–59%), and the C/P and N/P ratios in macroaggregates and medium-sized aggregates under the 3-year, plant-free condition, whereas NL-11 inoculation significantly enhanced TN (by 54–57%). Results from the simplified linear mixed-effects models indicated (Table 1) that experimental duration was the dominant factor regulating soil nutrient contents and their stoichiometric ratios (*p* < 0.001). Microbial inoculation significantly affected soil nutrient levels as well as the C/N and C/P ratios (*p* < 0.001). Plant presence significantly influenced soil total nitrogen and the C/N ratio (*p* < 0.05), and exerted highly significant effects on the C/P and N/P ratios (*p* < 0.001), whereas aggregate size showed no significant main effect. Significant interactive effects of Inoculants × Time and Inoculants × Plant were detected for SOC, TN, total phosphorus, and the C/P ratio (*p* < 0.01), among which the Inoculants × Plant interaction had the strongest effect on the N/P ratio (*p* < 0.001, Table 1).

**Figure 2:**
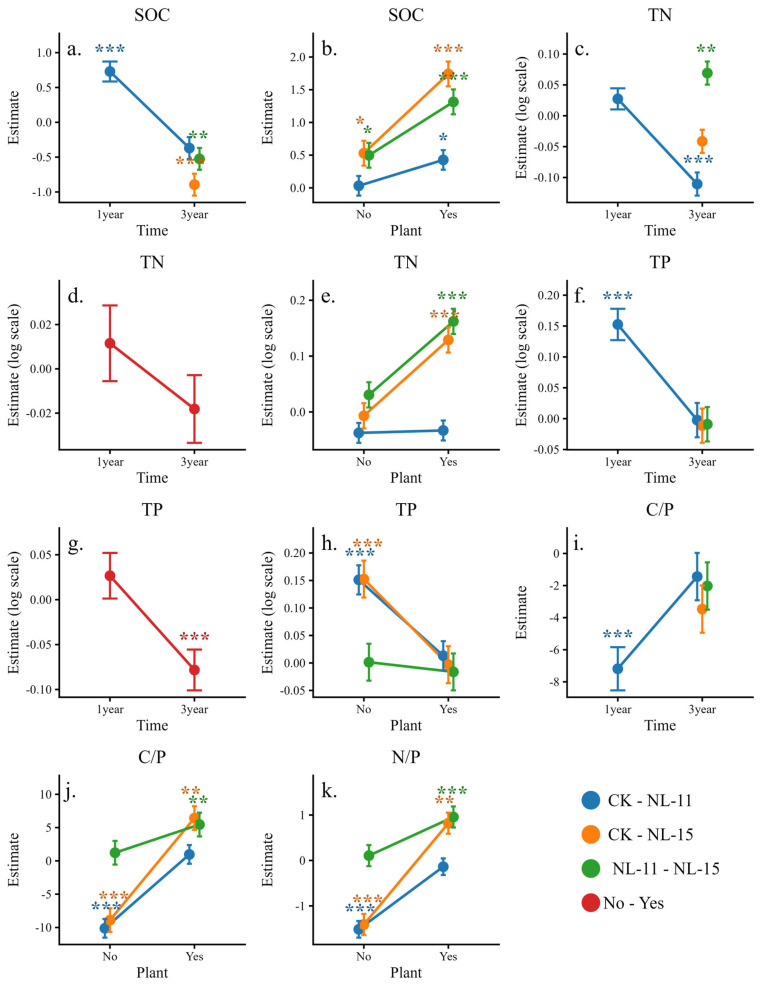
Simple-effects line plots illustrating significant two-way interactions for soil nutrient contents and their stoichiometric ratios. Values represent estimated marginal means (±SE) derived from simplified linear mixed-effects models. Some response variables were log-transformed prior to analysis. (**a**) SOC under the Time × Inoculants interaction; (**b**) SOC under the Plant × Inoculants interaction; (**c**) TN under the Time × Inoculants interaction; (**d**) TN under the Time × Plant interaction; (**e**) TN under the Plant × Inoculants interaction; (**f**) TP under the Time × Inoculants interaction; (**g**) TP under the Time × Plant interaction; (**h**) TP under the Plant × Inoculants interaction; (**i**) C/P under the Time × Inoculants interaction; (**j**) C/P under the Plant × Inoculants interaction; and (**k**) N/P under the Plant × Inoculants interaction. Statistical significance is indicated by asterisks (* *p* < 0.05, ** *p* < 0.01, *** *p* < 0.001).

Simple effects analysis revealed (Figure 2) that NL-11 decreased soil organic carbon and total phosphorus while increasing the C/P ratio in the 1-year experiment; however, the direction of its effects shifted in the 3-year experiment, with a significant increase in total nitrogen. The effects of NL-15 in the 3-year experiment were generally consistent with those of NL-11. Plant presence significantly modulated the effects of microbial inoculation. Under planted conditions, NL-15 reduced soil organic carbon and total nitrogen and reversed the responses of the C/P and N/P ratios from increases to decreases, whereas NL-11 reduced soil organic carbon only, and its stimulatory effects on stoichiometric ratios were weakened but not statistically significant. In addition, the Plant × Time interaction indicated that plant effects on total nitrogen and total phosphorus were reversed between experimental durations, with a significant enhancement of soil total phosphorus observed in the 3-year experiment. These results indicate that microbial inoculation alters soil nutrient stoichiometry in a strongly time- and plant-dependent manner.

### 3.3. Microbial Biomass Dynamics and Stoichiometric Stability

**Figure 3:**
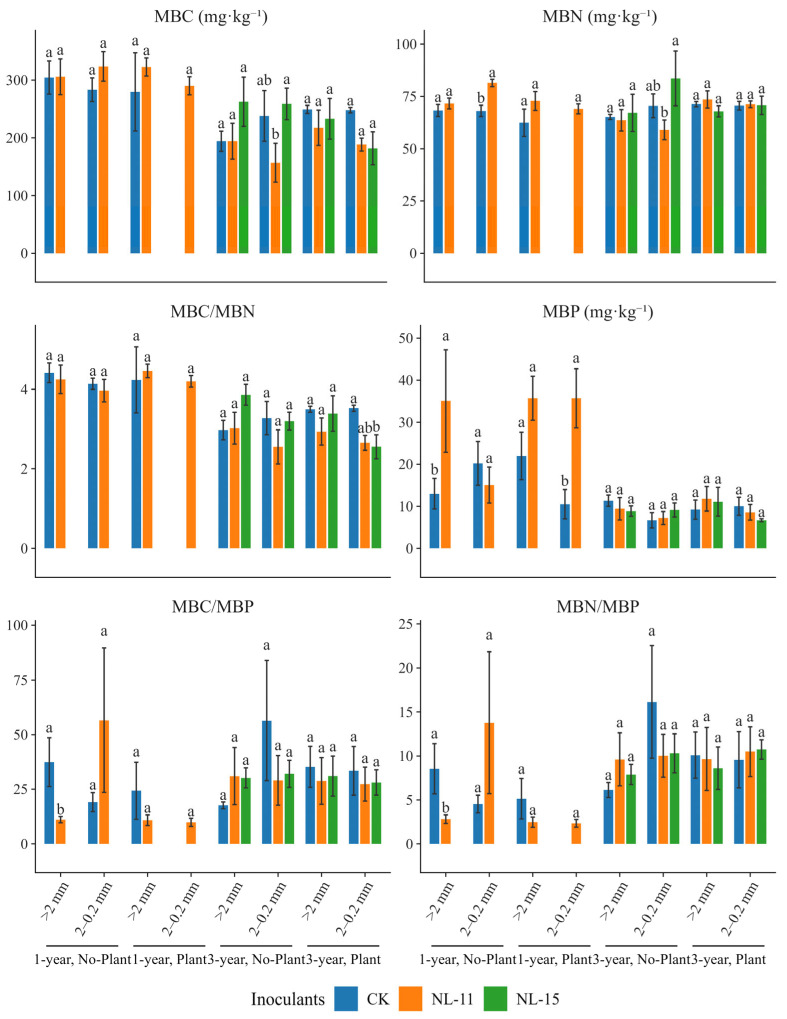
Responses of microbial biomass and its stoichiometric ratios to microbial inoculation. Bars represent means ± SE. Inoculants were as follows: CK, no inoculation; NL-11: treated with *Bacillus thuringiensis* inoculation; NL-15: treated with *Gongronella butleri* inoculation. Different lowercase letters indicate significant differences among inoculation treatments within the same Group (Time × Plant × Aggregate) based on Tukey-adjusted post hoc comparisons (*p* < 0.05). Percentage changes reported in the text were calculated relative to the control using estimated marginal means.

Compared with the control, NL-11 inoculation under the 1-year, No-plant, macroaggregate condition significantly increased MBP, with an increase of up to 170%, and significantly reduced the MBC/MBP and MBN/MBP ratios (Figure 3). Results from the simplified models indicated (Table 2) that time exerted highly significant effects on nearly all microbial biomass variables and their stoichiometric ratios (*p* < 0.001). In contrast, aggregate, plant, and inoculants treatment showed no significant main effects. The interaction between time and inoculation was the key factor driving changes in microbial biomass, with significant effects on MBC, MBN, and MBP (*p* < 0.05). The interaction between time and plant presence was significant only for MBN (*p* < 0.01).

**Figure 4:**
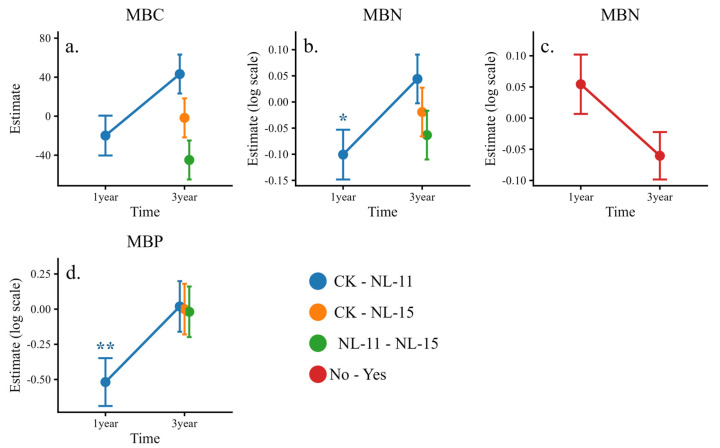
Simple-effects line plots illustrating significant two-way interactions for microbial biomass and its stoichiometric ratios. Values represent estimated marginal means (±SE) derived from simplified linear mixed-effects models. Some response variables were log-transformed prior to analysis. (**a**) MBC under the Time × Inoculants interaction; (**b**) MBN under the Time × Inoculants interaction; (**c**) MBN under the Time × Plant interaction; and (**d**) MBP under the Time × Inoculants interaction. Statistical significance is indicated by asterisks (* *p* < 0.05, ** *p* < 0.01).

Simple-effects analyses of the interaction effects indicated (Figure 4) that NL-11 exhibited a consistent time-dependent pattern for MBC and MBN, increasing both variables in the 1-year experiment but shifting to a decreasing effect in the 3-year experiment. In contrast, NL-15 tended to reduce MBC and slightly increase MBN, although these effects were not statistically significant. NL-11 significantly increased MBP only in the 1-year experiment, whereas neither NL-11 nor NL-15 showed significant effects on MBP in the 3-year experiment. Temporal reversals in microbial biomass responses suggest shifts in microbial nutrient limitation over time.

### 3.4. Enzyme Activities and Their Stoichiometric Ratios

**Figure 5:**
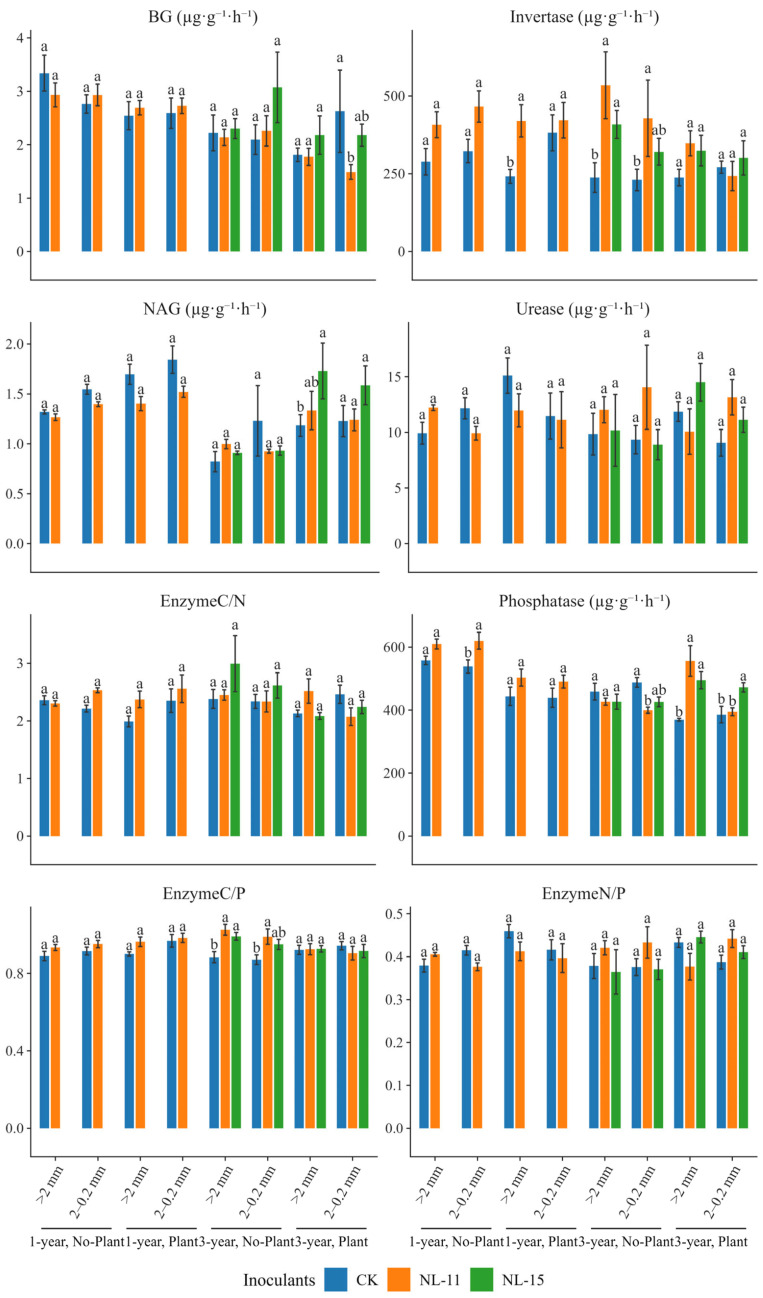
Responses of enzyme activities and their stoichiometric ratios to microbial inoculation. Bars represent means ± SE. Inoculants were as follows: CK, no inoculation; NL-11: treated with *Bacillus thuringiensis* inoculation; NL-15: treated with *Gongronella butleri* inoculation. Different lowercase letters indicate significant differences among inoculation treatments within the same Group (Time × Plant × Aggregate) based on Tukey-adjusted post hoc comparisons (*p* < 0.05). Percentage changes reported in the text were calculated relative to the control using estimated marginal means.

Compared with the control (Figure 5), both NL-11 and NL-15 inoculation significantly increased invertase activity (by 80–124%) under the 3-year, plant-free, macroaggregate condition. Under the 3-year condition with plant presence in macroaggregates, NL-15 significantly enhanced NAG activity (by 42%), and both inoculants significantly increased acid phosphatase activity (by 33–48%). Results of the simplified models indicated (Table 3) that Time, Plant, and Inoculants were the primary factors driving variations in enzyme activities and their stoichiometric ratios. Experimental duration significantly affected the activities of BG, NAG, Invertase, and acid phosphatase. Inoculants significantly influenced invertase activity, Acid Phosphatase activity, and the enzyme activity C/P ratio, whereas Plant significantly affected BG, NAG, and acid phosphatase activities, as well as the enzyme activity N/P and C/N ratios. Aggregate showed no significant effect on enzyme activities. Interaction effects revealed that the Inoculants × Plant interaction was the key driver regulating enzyme activity responses, with significant effects on NAG activity, acid phosphatase activity, and the enzyme activity C/P and N/P ratios (*p* < 0.05), and particularly strong effects on acid phosphatase activity (*p* < 0.001). In addition, the Time × Plant interaction significantly influenced Acid Phosphatase activity (*p* = 0.002; Table 3).

**Figure 6:**
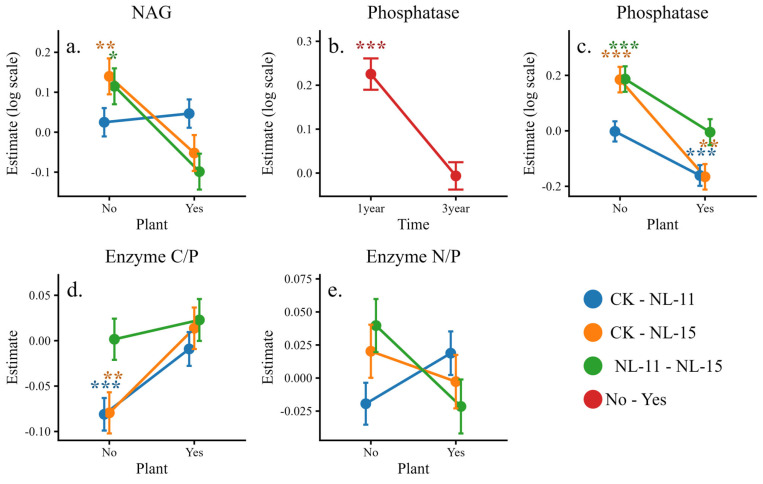
Simple-effects line plots illustrating significant two-way interactions for soil enzyme activities and their stoichiometric ratios. Values represent estimated marginal means (±SE) derived from simplified linear mixed-effects models. Some response variables were log-transformed prior to analysis. (**a**) NAG under the Plant × Inoculants interaction; (**b**) Phosphatase under the Time × Plant interaction; (**c**) Phosphatase under the Plant × Inoculants interaction; (**d**) Enzyme C/P under the Plant × Inoculants interaction; and (**e**) Enzyme N/P under the Plant × Inoculants interaction. Statistical significance is indicated by asterisks (* *p* < 0.05, ** *p* < 0.01, *** *p* < 0.001).

Further simple-effects analyses indicated (Figure 6) that plant presence significantly altered the direction of inoculation effects. Specifically, NL-15 decreased NAG activity under no-plant conditions but exhibited an increasing trend when plants were present. Both inoculants significantly enhanced acid phosphatase activity under planted conditions, whereas their stimulatory effects on enzyme activity C/P and N/P ratios were markedly weakened or reversed in the presence of plants, although these changes did not reach statistical significance. Changes in enzyme activities and stoichiometric ratios reflect microbial adjustments in nutrient acquisition strategies.

### 3.5. Stoichiometric Coupling Among Soil Nutrients, Microbial Biomass, and Enzyme Allocation

**Figure 7:**
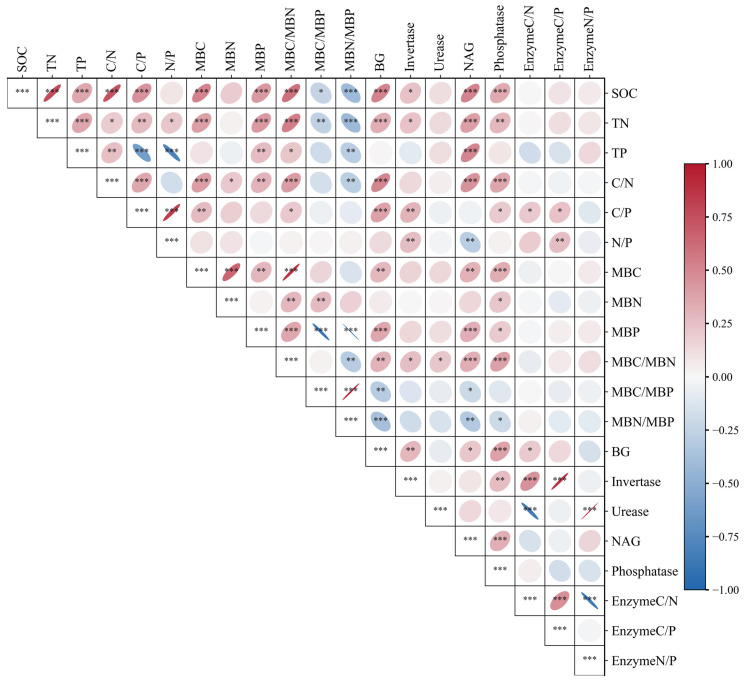
Heatmap of correlation coefficients among soil properties, microbial biomass, and enzyme activity indicators. Ellipses indicate the strength and direction of correlations among variables, with colors ranging from blue (negative correlations) to red (positive correlations). Statistical significance is indicated by asterisks (* *p* < 0.05, ** *p* < 0.01, *** *p* < 0.001).

Correlation analysis revealed a strongly coupled stoichiometric relationship among soil nutrients, microbial biomass, and extracellular enzyme activities (Figure 7). SOC showed significant positive correlations with TN, TP, and soil stoichiometric ratios (C/N and C/P). SOC was also positively related to MBC, MBP, and MBC/MBN, but negatively related to MBC/MBP and MBN/MBP. TN was positively correlated with SOC, TP, and soil stoichiometric ratios, and it also showed positive relationships with MBC, MBP, and MBC/MBN. TP was positively associated with SOC, TN, and C/N, while it was negatively correlated with C/P and N/P. MBC, MBN, MBP, and their stoichiometric ratios were generally correlated with soil nutrient indicators. In particular, MBC/MBN showed positive correlations with several soil nutrients and enzyme activities, whereas MBC/MBP and MBN/MBP were negatively related to most nutrient and enzyme variables. All extracellular enzymes (BG, invertase, NAG, and ACP) were positively correlated with each other and were overall positively associated with SOC, TN, and their stoichiometric ratios. In contrast, urease activity was negatively correlated with the enzyme activity C/N ratio but positively correlated with the enzyme activity N/P ratio. At the level of enzyme stoichiometry, the enzyme activity C/N ratio was significantly positively correlated with the C/P ratio, whereas the enzyme activity N/P ratio was significantly negatively correlated with the C/N ratio, reflecting coordination and trade-offs among different nutrient acquisition strategies.

## 4. Discussion

In this study, we used linear mixed-effects models to examine how soil C, N, and P stoichiometric characteristics respond to multiple interacting factors. The results indicate that experimental duration is a key driver of changes in soil nutrients, microbial biomass, and enzyme activities [37], highlighting the strong time dependence of microbial inoculation effects. In our experiment, the NL-11 treatment promoted organic carbon decomposition and phosphorus release after 1 year, while it significantly increased organic carbon and total nitrogen after 3 years (Figure 2). This pattern likely reflects two concurrent processes following functional microbial inoculation. First, the introduced microorganisms compete with native communities and support their own growth by decomposing organic matter, which leads to short-term reductions in SOC, TN, and TP [38,39]. Second, as time progresses and the microbial community approaches a more stable state, the inoculants improve soil resource availability through carbon fixation, nitrogen fixation, and phosphorus activation [4,5,6,7]. However, due to early nutrient consumption and the absence of additional organic matter inputs, soil nutrient pools had not fully recovered to initial levels. This may explain why overall soil nutrient contents at the 3-year scale were lower than those observed after 1 year. Statistical analysis revealed no significant effects of aggregate size on soil nutrients, microbial biomass, or enzyme activities. It should be noted, however, that our observations were based on macro-aggregate (>2 mm) and meso-aggregate (0.25–2 mm), while microaggregates (<0.25 mm) were not included in this study. Consequently, the findings characterize the biochemical properties specifically within the boundary of larger aggregate fractions.

Analysis of aggregate size distribution and MWD revealed that differences in aggregate distribution primarily emerged under plant-present conditions, whereas minimal structural changes were observed in treatments without plants. Furthermore, the specific aggregate fractions affected differed between the 1-year and 3-year experiments, suggesting that the alterations in soil structure were not a result of uniform modification, but rather a gradual redistribution among size classes. These findings indicate that changes in soil physical structure may not originate from direct inoculation-driven aggregation processes, but instead appear to be mediated by plant–microbe interactions [40,41].

Soil microorganisms obtain resources for biomass production by secreting extracellular enzymes [42,43], and their growth is constrained by soil nutrient availability, especially nitrogen and phosphorus [44]. Our results show that after microbial inoculation, microbial biomass C, N, and P increased at the 1-year stage but declined at the 3-year stage (Figure 2). This pattern differs from previous studies reporting consistent increases in microbial biomass C and N after inoculation and their positive relationships with soil C and N availability [45,46]. A possible explanation is that, shortly after inoculation, rapid decomposition of soil organic matter releases large amounts of nutrients, which stimulates microbial biomass accumulation. Over longer periods, the microbial community becomes more stable, while declining soil nutrient availability imposes nutrient limitation on microorganisms, leading to a reduction in biomass. Microbial biomass C, N, and P stoichiometry is a key indicator of soil nutrient cycling [44,47]. Our results indicate that microbial inoculation had no significant effect on microbial stoichiometric ratios. This finding is consistent with ecological stoichiometry theory, which suggests that microbial biomass stoichiometry remains relatively stable, and that microorganisms respond to soil nutrient limitation mainly by adjusting enzyme activities rather than changing their biomass composition [48].

Plant–microbe interactions play key roles in a series of nutrient cascades [49]. The rhizosphere is one of the most complex ecosystems on Earth and also a hotspot of microbial activity, with strong influence on soil nutrient cycling and accumulation [35]. Soil enzyme activity is a sensitive indicator of subtle changes in the soil environment, and enzyme stoichiometric ratios reflect microbial resource-use strategies [18,44]. The significant interaction between plant presence and microbial inoculation indicates that rhizosphere conditions regulate the functional effects of inoculants. The combined presence of plants and microbial inoculation increased organic matter consumption, which reflects the high demand for energy and nutrients by both plants and microorganisms. In contrast, the time-dependent reversal of plant effects (Figure 2) suggests that microbial inoculation may contribute to system stabilization through plant–microbe interactions over the long term. Increases in enzyme activities, especially phosphatase (Figure 6), together with declines in soil C/P and N/P ratios (Figure 2), indicate that plant–microbe interactions alleviated soil nutrient limitation. This finding agrees with previous studies showing that inoculants promote the production of key enzymes involved in phosphorus cycling [50]. The overall effect direction of NL-15 and NL-11 was similar, but some differences were observed. NL-15 showed a stronger regulatory effect on enzyme activities, which may be related to differences in physiological functions and ecological strategies between the two inoculants. Additionally with respect to the soil N/P ratio (Figure 2k), the results indicate that plant presence modified inoculant responses in a strain-specific manner. NL-15 exhibited opposite trends under plant-present versus plant-absent conditions, shifting from an increase to a decrease in N/P ratio, whereas the N/P-enhancing effect of NL-11 was weakened when plants were present. These patterns suggest that rhizosphere conditions influenced how inoculants expressed their functional effects. Although *A. fruticosa* is a nitrogen-fixing legume and may contribute biologically fixed nitrogen to the soil system, the overall N/P ratio did not show a uniform increase under plant presence. Instead, N/P tended to decline or showed a reduced increasing trend when microbial inoculation and plant presence co-occurred. This observation implies that plant–microbe interactions may have redirected nutrient dynamics toward alleviating phosphorus limitation rather than simply amplifying nitrogen inputs.

Correlation analysis further clarified the coordinated relationships among soil C, N, P pools and their stoichiometric ratios. The significant positive correlations between SOC and TN, TP, as well as C/N and C/P ratios indicate that these nutrient variables tended to vary in a coordinated manner across treatments. At the microbial level, the microbial biomass C/N ratio was positively correlated with soil nutrients and enzyme activities, while it was negatively correlated with microbial biomass C/P and N/P ratios. These patterns correspond to shifts in microbial nutrient allocation status under different nutrient conditions [44]. Phosphatase activity did not show a significant correlation with microbial biomass C/P (Figure 7). The absence of this association may be related to the broad substrate specificity of soil enzymes as well as potential changes in microbial community composition following inoculation [51,52]. Positive correlations among BG, invertase, NAG, and acid phosphatase indicate concurrent variation in enzyme activities, which may reflect coordinated functional responses within the soil microbial system [42,48]. It should be noted that these correlations represent statistical associations among functional indicators rather than direct mechanistic evidence.

## 5. Conclusions

This study analyzed the effects of NL-11 and NL-15 inoculants on soil aggregate stability and the distribution of aggregate size fractions. Our results showed that microbial inoculation significantly altered the soil physical structure in the presence of plants, with plant–microbe interactions markedly enhancing aggregate stability. We applied linear mixed-effects models to examine how functional microbial inoculation interacts with experimental duration, plant presence, and aggregate size to shape soil nutrient status, microbial biomass, and enzyme-related stoichiometric patterns. The results indicate that time was the dominant factor regulating soil C–N–P dynamics, while inoculation effects differed between early and later stages. In the short term, NL-11 promoted carbon turnover and phosphorus release, while in the long term, it facilitated the accumulation of carbon and nitrogen. Differences between the two inoculants were also evident: NL-11 mainly influenced soil nutrient availability and microbial biomass responses, while NL-15 exerted a stronger regulatory effect on enzyme activities. Plant presence further modified inoculation responses, underscoring the importance of plant–microbe interactions in soil nutrient regulation. Changes in microbial biomass and enzyme stoichiometric ratios suggest that microorganisms primarily adjust enzyme investment related to carbon, nitrogen, and phosphorus acquisition to cope with environmental constraints. Overall, functional microbial inoculation influences soil nutrient allocation and C–N–P cycling mainly through microbial and enzymatic processes, providing insight into its potential role in the long-term restoration of degraded soils.

## 6. Study Limitations

This study assessed microbial responses to inoculation using microbial biomass and extracellular enzyme activities as functional indicators. These measurements reflect integrated microbial processes but do not provide direct molecular evidence of microbial community composition, inoculant persistence, or functional gene dynamics. Therefore, mechanistic interpretations should be considered functionally based rather than taxonomically resolved. In addition, the pot-based design may not fully represent field-scale soil complexity. Future studies integrating molecular approaches and field validation would help further clarify the mechanisms underlying aggregate-scale nutrient stoichiometric responses.

## Figures and Tables

**Table 1:** Significance tests of main effects and two-way interactions of microbial inoculation, experimental duration, plant presence, and aggregate size on soil nutrient contents and their stoichiometric ratios, based on simplified linear mixed-effects models.

	SOC	TN	TP	C/N	C/P	N/P
T	<0.001	<0.001	<0.001	<0.001	<0.001	0.546
I	<0.001	0.004	<0.001	<0.001	<0.001	<0.001
P	0.376	0.034	0.095	0.02	<0.001	<0.001
A	0.118	0.444	0.522	0.554	0.766	0.677
T × I	<0.001	<0.001	<0.001	0.138	0.005	0.291
T × P	0.08	<0.001	0.015	0.323	0.294	0.507
T × A	0.221	0.962	0.383	0.688	0.331	0.175
I × P	<0.001	<0.001	0.001	0.082	<0.001	<0.001
I × A	0.911	0.664	0.978	0.77	0.931	0.886
P × A	0.563	0.159	0.640	0.141	0.483	0.896

Note: Values represent *p*-values from Type III analysis of variance (F-tests) based on simplified linear mixed-effects models including only main effects and two-way interactions. I: Inoculants; P: Plant; T: Time; A: Aggregate. T × I: interaction between Inoculants and Time; T × P: interaction between Plant and Time; T × A: interaction between Time and Aggregate; I × P: interaction between Inoculants and Plant; I × A: interaction between Inoculants and Aggregate; P × A: interaction between Aggregate and Plant.

**Table 2:** Significance tests of main effects and two-way interactions of microbial inoculation, experimental duration, plant presence, and aggregate size on microbial biomass and its stoichiometric ratios, based on simplified linear mixed-effects models.

	MBC	MBN	MBP	MBC/MBN	MBC/MBP	MBN/MBP
T	<0.001	0.805	<0.001	<0.001	0.004	<0.001
I	0.342	0.229	0.147	0.1	0.364	0.746
P	0.215	0.175	0.205	0.496	0.072	0.091
A	0.282	0.228	0.125	0.056	0.744	0.318
T × I	0.027	0.01	0.036	0.112	0.883	0.548
T × P	0.166	0.003	0.419	0.643	0.084	0.094
T × A	0.848	0.783	0.927	0.878	0.569	0.447
I × P	0.061	0.082	0.378	0.071	0.815	0.935
I × A	0.661	0.263	0.624	0.153	0.566	0.361
P × A	0.356	0.165	0.800	0.781	0.292	0.214

Note: Values represent *p*-values from Type III analysis of variance (F-tests) based on simplified linear mixed-effects models including only main effects and two-way interactions. I: Inoculants; P: Plant; T: Time; A: Aggregate. T × I: interaction between Inoculants and Time; T × P: interaction between Plant and Time; T × A: interaction between Time and Aggregate; I × P: interaction between Inoculants and Plant; I × A: interaction between Inoculants and Aggregate; P × A: interaction between Aggregate and Plant.

**Table 3:** Significance tests of main effects and two-way interactions of microbial inoculation, experimental duration, plant presence, and aggregate size on enzyme activities and their stoichiometric ratios, based on simplified linear mixed-effects models.

	BG	Invertase	Urease	NAG	Phosphatase	EnzymeC/N	EnzymeC/P	EnzymeN/P
T	<0.001	0.010	0.408	<0.001	<0.001	0.994	0.707	0.910
I	0.068	<0.001	0.764	0.053	0.003	0.202	0.002	0.840
P	0.011	0.199	0.035	<0.001	0.005	0.005	0.736	0.003
A	0.643	0.961	0.246	0.237	0.282	0.571	0.681	0.386
T × I	0.321	0.784	0.114	0.067	0.111	0.200	0.450	0.053
T × P	0.920	0.368	0.627	0.477	0.002	0.825	0.058	0.396
T × A	0.537	0.052	0.425	0.509	0.295	0.169	0.091	0.380
I × P	0.803	0.177	0.064	0.019	<0.001	0.058	0.019	0.049
I × A	0.760	0.166	0.703	0.314	0.101	0.642	0.503	0.549
P × A	0.818	0.649	0.271	0.278	0.147	0.199	0.301	0.331

Note: Values represent *p*-values from Type III analysis of variance (F-tests) based on simplified linear mixed-effects models including only main effects and two-way interactions. I: Inoculants; P: Plant; T: Time; A: Aggregate. T × I: interaction between Inoculants and Time; T × P: interaction between Plant and Time; T × A: interaction between Time and Aggregate; I × P: interaction between Inoculants and Plant; I × A: interaction between Inoculants and Aggregate; P × A: interaction between Aggregate and Plant.

## Data Availability

The original contributions presented in this study are included in the article/Supplementary Material. Further inquiries can be directed to the corresponding author.

## Supplementary Materials

The following supporting information can be downloaded at: https://www.mdpi.com/article/10.3390/microorganisms14030583/s1, Figure S1: Effects of microbial inoculation on soil aggregate size distribution and mean weight diameter (MWD) under varying experimental durations and plant presence conditions; Table S1: Statistical results of non-parametric tests for soil aggregate fractions and MWD.

## References

[1] Poria V., Dębiec-Andrzejewska K., Fiodor A., Lyzohub M., Ajijah N., Singh S., Pranaw K. (2022). Plant Growth-Promoting Bacteria (PGPB) Integrated Phytotechnology: A Sustainable Approach for Remediation of Marginal Lands. Front. Plant Sci..

[2] Guo Y., Bi Y., Li P., Christie P. (2024). Role of an Arbuscular Mycorrhizal Fungus in Vegetation Restoration as Indicated by Bacterial Diversity and Microbial Metabolic Limitation in Soil Underlying Moss Biocrusts. Int. Biodeterior. Biodegrad..

[3] Falkowski P.G., Fenchel T., Delong E.F. (2008). The Microbial Engines That Drive Earth’s Biogeochemical Cycles. Science.

[4] Elhaissoufi W., Ghoulam C., Barakat A., Zeroual Y., Bargaz A. (2022). Phosphate Bacterial Solubilization: A Key Rhizosphere Driving Force Enabling Higher P Use Efficiency and Crop Productivity. J. Adv. Res..

[5] Aasfar A., Bargaz A., Yaakoubi K., Hilali A., Bennis I., Zeroual Y., Kadmiri I.M. (2021). Nitrogen Fixing *Azotobacter* Species as Potential Soil Biological Enhancers for Crop Nutrition and Yield Stability. Front. Microbiol..

[6] Sinsabaugh R.L., Lauber C.L., Weintraub M.N., Ahmed B., Allison S.D., Crenshaw C., Contosta A.R., Cusack D., Frey S., Gallo M.E. (2008). Stoichiometry of Soil Enzyme Activity at Global Scale. Ecol. Lett..

[7] Allison S.D., Vitousek P.M. (2005). Responses of Extracellular Enzymes to Simple and Complex Nutrient Inputs. Soil Biol. Biochem..

[8] Bai Z., Caspari T., Gonzalez M.R., Batjes N.H., Mäder P., Bünemann E.K., de Goede R., Brussaard L., Xu M., Ferreira C.S.S. (2018). Effects of Agricultural Management Practices on Soil Quality: A Review of Long-Term Experiments for Europe and China. Agric. Ecosyst. Environ..

[9] Six J., Bossuyt H., Degryze S., Denef K. (2004). A History of Research on the Link between (Micro)Aggregates, Soil Biota, and Soil Organic Matter Dynamics. Soil Tillage Res..

[10] Rillig M.C., Mummey D.L. (2006). Mycorrhizas and Soil Structure. New Phytol..

[11] Six J., Elliott E.T., Paustian K. (2000). Soil Macroaggregate Turnover and Microaggregate Formation: A Mechanism for C Sequestration under No-Tillage Agriculture. Soil Biol. Biochem..

[12] Rillig M.C., Aguilar-Trigueros C.A., Bergmann J., Verbruggen E., Veresoglou S.D., Lehmann A. (2015). Plant Root and Mycorrhizal Fungal Traits for Understanding Soil Aggregation. New Phytol..

[13] Bach E.M., Baer S.G., Meyer C.K., Six J. (2010). Soil Texture Affects Soil Microbial and Structural Recovery during Grassland Restoration. Soil Biol. Biochem..

[14] Zhu Y., Guo B., Liu C., Lin Y., Fu Q., Li N., Li H. (2021). Soil Fertility, Enzyme Activity, and Microbial Community Structure Diversity among Different Soil Textures under Different Land Use Types in Coastal Saline Soil. J. Soils Sediments.

[15] Xiong J., Shao X., Yuan H., Liu E., Xu H., Wu M., Xiong J., Shao X., Yuan H., Liu E. (2023). Effect of Human Reclamation and *Spartina alterniflora* Invasion on C-N-P Stoichiometry in Plant Organs across Coastal Wetlands over China. Plant Soil.

[16] Li W., Liu Y., Zheng H., Wu J., Yuan H., Wang X., Xie W., Qin Y., Zhu H., Nie X. (2023). Complex Vegetation Patterns Improve Soil Nutrients and Maintain Stoichiometric Balance of Terrace Wall Aggregates over Long Periods of Vegetation Recovery. CATENA.

[17] Wang G., Xiao H., Xin Z., Luo F., Jin Y., Liu M., Li J. (2024). Changes in Plant-Soil-Microbe C-N-P Contents and Stoichiometry during Poplar Shelterbelt Degradation. CATENA.

[18] Cleveland C.C., Liptzin D. (2007). C:N:P Stoichiometry in Soil: Is There a “Redfield Ratio” for the Microbial Biomass?. Biogeochemistry.

[19] Xu X., Thornton P.E., Post W.M. (2013). A Global Analysis of Soil Microbial Biomass Carbon, Nitrogen and Phosphorus in Terrestrial Ecosystems. Glob. Ecol. Biogeogr..

[20] Yang W., Zhao Y., Yang Y., Zhang M., Mao X., Guo Y., Li X., Tao B., Qi Y., Ma L. (2022). Co-Application of Biochar and Microbial Inoculants Increases Soil Phosphorus and Potassium Fertility and Improves Soil Health and Tomato Growth. J. Soils Sediments.

[21] Luo Z., Han H., Yao H., Yan G., Bai J., Shi L., Pei X., Li J., Li Q., Luo Z. (2024). Effects of Artificially Modified Microbial Communities on the Root Growth and Development of Tall Fescue in Nutrient-Poor Rubble Soil. Plants.

[22] Feng Q., Cao S., Liao S., Wassie M., Sun X., Chen L., Xie Y. (2023). Fusarium Equiseti-Inoculation Altered Rhizosphere Soil Microbial Community, Potentially Driving Perennial Ryegrass Growth and Salt Tolerance. Sci. Total Environ..

[23] Cui H., Zhu H., Shutes B., Rousseau A.N., Feng W.D., Hou S.N., Ou Y., Yan B.X. (2023). Soil Aggregate-Driven Changes in Nutrient Redistribution and Microbial Communities after 10-Year Organic Fertilization. J. Environ. Manag..

[24] Wu Y., Zhang J., Guo X. (2017). An Indigenous Soil Bacterium Facilitates the Mitigation of Rocky Desertification in Carbonate Mining Areas. Land Degrad. Dev..

[25] Li C., Shi Y., Jia Z., Tang Y., Lin J., Liu X., Zhang J., Müller C. (2025). Microbial Inoculants Modify the Functions of Resident Soil Microbes to Expedite the Field Restoration of the Abandoned Mine. Land Degrad. Dev..

[26] Wang G., Deng H., Nie L., Wu Y., Zhang J. (2018). Corrosion mechanism of limestone by *Gongronella butleri* NL-15. Chin. J. Appl. Environ. Biol..

[27] Tian S., Zhu B., Yin R., Wang M., Jiang Y., Zhang C., Li D., Chen X., Kardol P., Liu M. (2022). Organic Fertilization Promotes Crop Productivity through Changes in Soil Aggregation. Soil Biol. Biochem..

[28] Harris D., Horwáth W.R., van Kessel C. (2001). Acid Fumigation of Soils to Remove Carbonates Prior to Total Organic Carbon or CARBON-13 Isotopic Analysis. Soil Sci. Soc. Am. J..

[29] Thomas G.W. (1982). Exchangeable Cations. Methods of Soil Analysis.

[30] Vance E.D., Brookes P.C., Jenkinson D.S. (1987). An Extraction Method for Measuring Soil Microbial Biomass C. Soil Biol. Biochem..

[31] Brookes P.C., Landman A., Pruden G., Jenkinson D.S. (1985). Chloroform Fumigation and the Release of Soil Nitrogen: A Rapid Direct Extraction Method to Measure Microbial Biomass Nitrogen in Soil. Soil Biol. Biochem..

[32] Brookes P.C., Powlson D.S., Jenkinson D.S. (1982). Measurement of Microbial Biomass Phosphorus in Soil. Soil Biol. Biochem..

[33] Tabatabai M.A. (2003). Soil Enzymes. Methods of Soil Analysis.

[34] Guan S.Y., Zhang D., Zhang Z. (1986). Soil Enzymes and Its Methods.

[35] Ekenler M., Tabatabai M.A. (2002). β-Glucosaminidase Activity of Soils: Effect of Cropping Systems and Its Relationship to Nitrogen Mineralization. Biol. Fertil. Soils.

[36] Ren Y., Wang Y., Zhang X., Liu X., Liu P., Chen L., Ren Y., Wang Y., Zhang X., Liu X. (2024). Enzymatic Stoichiometry Reveals the Metabolic Limitations of Soil Microbes under Nitrogen and Phosphorus Addition in Chinese Fir Plantations. Microorganisms.

[37] Feng J., Chen L., Xia T., Ruan Y., Sun X., Wu T., Zhong Y., Shao X., Tang Z. (2023). Microbial Fertilizer Regulates C:N:P Stoichiometry and Alleviates Phosphorus Limitation in Flue-Cured Tobacco Planting Soil. Sci. Rep..

[38] Kuzyakov Y., Blagodatskaya E. (2015). Microbial Hotspots and Hot Moments in Soil: Concept & Review. Soil Biol. Biochem..

[39] Englander A.C., Douds D.D., Mallory E.B. (2016). On-Farm Produced Microbial Soil Inoculant Effects on Bread Wheat (*Triticum aestivum*) Production. Biol. Agric. Hortic..

[40] Li J., Che Y., Chen S., Liu M., Diao M., Yang C., Jia W. (2025). *Bacillus tropicus* YJ33 and *Medicago sativa* L. Synergistically Enhance Soil Aggregate Stability in Saline–Alkali Environments. Microorganisms.

[41] Mueller C.W., Baumert V., Carminati A., Germon A., Holz M., Kögel-Knabner I., Peth S., Schlüter S., Uteau D., Vetterlein D. (2024). From Rhizosphere to Detritusphere—Soil Structure Formation Driven by Plant Roots and the Interactions with Soil Biota. Soil Biol. Biochem..

[42] Martiny J.B.H., Bohannan B.J.M., Brown J.H., Colwell R.K., Fuhrman J.A., Green J.L., Horner-Devine M.C., Kane M., Krumins J.A., Kuske C.R. (2006). Microbial Biogeography: Putting Microorganisms on the Map. Nat. Rev. Microbiol..

[43] Manzoni S., Trofymow J.A., Jackson R.B., Porporato A. (2010). Stoichiometric Controls on Carbon, Nitrogen, and Phosphorus Dynamics in Decomposing Litter. Ecol. Monogr..

[44] Sterner R.W., Elser J.J. (2002). Ecological Stoichiometry: The Biology of Elements from Molecules to the Biosphere.

[45] Han Y., Yuan G., Yang X., Fang L., Liang Y., Zhou B., Wei Z. (2025). Arbuscular Mycorrhizal Fungi Enhance Soil Nutrient Cycling by Regulating Soil Bacterial Community Structures in Mango Orchards with Different Soil Fertility Rates. Front. Microbiol..

[46] Rehman M.M.U., Zhao L., Khattak S., Xiao Y.L., Iqbal A., Khan W., Abrar M., Cheng Z.G., Li S.S., Batool A. (2025). Amplification Effects of AM Fungus and Rhizobacteria on Carbon Efficiency in Wheat-Soil System under Drought Stress via Priming Rhizosphere Activities. Appl. Soil Ecol..

[47] Li J., Liu Y., Hai X., Shangguan Z., Deng L. (2019). Dynamics of Soil Microbial C:N:P Stoichiometry and Its Driving Mechanisms Following Natural Vegetation Restoration after Farmland Abandonment. Sci. Total Environ..

[48] Schimel J., Balser T.C., Wallenstein M. (2007). Microbial Stress-Response Physiology and Its Implications for Ecosystem Function. Ecology.

[49] Jones D.L., Hinsinger P. (2008). The Rhizosphere: Complex by Design. Plant Soil.

[50] Angelina E., Papatheodorou E.M., Demirtzoglou T., Monokrousos N., Angelina E., Papatheodorou E.M., Demirtzoglou T., Monokrousos N. (2020). Effects of *Bacillus Subtilis* and *Pseudomonas Fluorescens* Inoculation on Attributes of the Lettuce (*Lactuca sativa* L.) Soil Rhizosphere Microbial Community: The Role of the Management System. Agronomy.

[51] Wei S., Ding S., Lin H., Li Y., Zhang E., Liu T., Duan X. (2024). Microbial and Enzymatic C:N:P Stoichiometry Are Affected by Soil C:N in the Forest Ecosystems in Southwestern China. Geoderma.

[52] Mooshammer M., Wanek W., Zechmeister-Boltenstern S., Richter A. (2014). Stoichiometric Imbalances between Terrestrial Decomposer Communities and Their Resources: Mechanisms and Implications of Microbial Adaptations to Their Resources. Front. Microbiol..

