# Supplementary material for: Effects of Microbial Inoculants on Carbon, Nitrogen, and Phosphorus Stoichiometry of Soil Aggregates
Source: Microorganisms. 2026 Mar 4;14(3):583. doi: 10.3390/microorganisms14030583 (PMC13029648; doi:10.3390/microorganisms14030583)
Supplement: Supplementary file 1 [file microorganisms-14-00583-s001.zip › microorganisms-4111096-supplementary.pdf]

Supplementary Materials: Effects of Microbial Inoculants on Carbon, Nitrogen, and Phosphorus Stoichiometry of Soil Aggregates

Rengui Xue [1], Chong Li [2], Xin Liu [1], Xuanran Yu [1], Ying Chen [1], Yue Chen [1] and Jinchi Zhang [1,*]

1 Co-Innovation Center for Sustainable Forestry in Southern China of Jiangsu Province, Key Laboratory of Soil and Water Conservation and Ecological Restoration of Jiangsu Province, Nanjing Forestry University, Nanjing 210037, China; njfuxrg@njfu.edu.cn (R.X.) (ORCID: 0009-0002-1674-3530); liuxinswc@njfu.edu.cn (X.L.) (OR-CID: 0000-0001-8641-7170); yuxuanran@njfu.edu.cn (X.Y.) (ORCID: 0009-0004-4296-3355); ccchen71@njfu.edu.cn (Y.C.); 19860915971@njfu.edu.cn (Y.C.)

2 Department of Renewable Resources, University of Alberta, Edmonton, AB T6G 2E3, Canada; cli5104@njfu.edu.cn (ORCID: 0000-0001-9330-5396)

* Correspondence: zhang8811@njfu.edu.cn (ORCID: 0000-0002-0517-7214); Tel.: + 86 13809000518

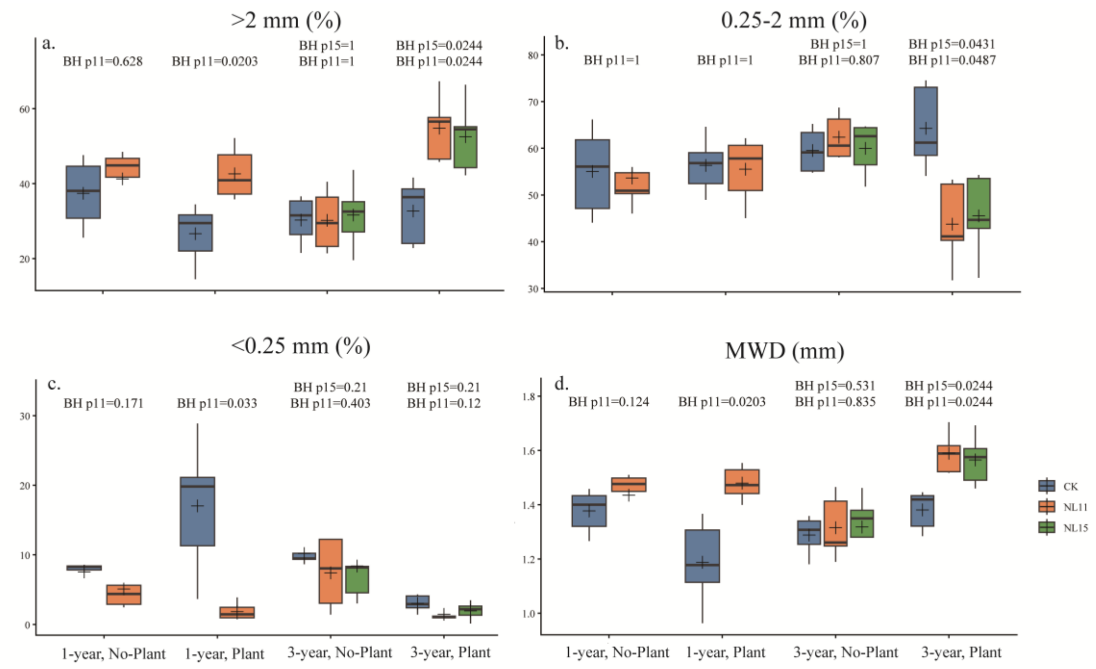

Figure S1. Effects of microbial inoculation on soil aggregate size distribution and mean weight diameter (MWD) under varying experimental durations and plant presence conditions. Subplots show the proportions of **(a)** >2 mm aggregates, **(b)** 0.25–2 mm aggregates, and **(c)** <0.25 mm aggregates, as well as **(d)** the mean weight diameter (MWD). Treatments include control (CK), NL11, and NL15 (NL15 was applied only in 3-year experiments) across 1-year and 3-year durations, under No-Plant and Plant conditions. In the boxplots, the horizontal line within the box represents the median, the '+' sign indicates the mean, the top and bottom of the box represent the 75th and 25th percentiles, respectively, and the whiskers extend to 1.5 times the interquartile range. The text annotations above the boxplots display the *p*-values from Wilcoxon rank-sum tests, adjusted using the Benjamini–Hochberg (BH) procedure, for comparisons against the CK treatment within each Time × Plant combination. Specifically, "**BH p11**" indicates the adjusted *p*-value for the comparison between NL11 and CK, and "**BH p15**" indicates the adjusted *p*-value for the comparison between NL15 and CK.

Table S1. Statistical results of non-parametric tests for soil aggregate fractions and MWD.

	Time	Plant	global	NL11 vs. CK	NL15 vs. CK
>2 mm (%)	1-year	No-Plant	0.628	0.628	—
		Plant	0.015	0.020	—
	3-year	No-Plant	0.990	1.000	1.000
		Plant	0.015	0.024	0.024
0.25–2 mm (%)	1-year	No-Plant	1.000	1.000	—
		Plant	1.000	1.000	—
	3-year	No-Plant	1.000	0.807	1.000
		Plant	0.041	0.049	0.043
<0.25 mm (%)	1-year	No-Plant	0.171	0.171	—
		Plant	0.033	0.033	—
	3-year	No-Plant	0.340	0.403	0.210
		Plant	0.171	0.120	0.210
MWD	1-year	No-Plant	0.124	0.124	—
		Plant	0.016	0.020	—
	3-year	No-Plant	0.811	0.835	0.531
		Plant	0.016	0.024	0.024

Note: All P values in the table have been adjusted and are presented as P_{adj}. P_{adj} values represent *p-values* adjusted by the Benjamini–Hochberg procedure. Kruskal–Wallis tests were used for global comparisons, followed by Wilcoxon rank-sum tests for pairwise comparisons against the control (CK). '—': not applicable (NL15 was not included in the 1-year experiment).
